# Missouri

**Published:** 1899-04

**Authors:** W. A. Heck

**Affiliations:** Secretary


### SOCIETY PROCEEDINGS.

### MISSOURI VALLEY VETERINARY ASSOCIATION.

THE nineteenth regular meeting was held in Kansas City, Mo., on Monday evening, February 27, 1899, at the Kansas City Veterinary College. On account of the absence of the President, the Association was called to order by Vice-President Robert C. Moore. The following members were present: Drs. John Forbes, James S. Kelly, John B. Wright, James L. Otterman, E. J. Netherton, William A. Heck, St. Joseph, Mo.; Drs. R. C. Moore, J. B. Black, S. Stewart, B. F. Kaupp, C. J. Sihler, F. C. McCurdy, James S. Buckley, Kansas City, Mo.; Drs. E. H. Biart and John Ernst, Leavenworth, Kansas; Dr. J. H. Cock, Ottawa, Kansas. The following visitors were present: Drs. H. B. Chaney, C. H. Canfield, R. T. W. Carnachan, H. H. George, Albert Long, D. W. Patton, W. R. Cooper, and A. C. Ewart, of Kansas City, Mo.; James Wilson, J. E. Blackwell, and Thomas H. Ripley, of St. Joseph, Mo.; O. Nickson, Cameron, Mo.; G. R. Conrad, Sabetha, Kansas; W. N. Hobbs, Holton, Kansas; W. A. Porter, Sedalia, Mo. There were also present about thirty veterinary students and several laymen.

The censors reported favorably upon the applications for membership of James L. Otterman, W. N. Hobbs, and W. Ross Cooper, and upon motion the rules were suspended and the Secretary instructed to cast the vote of the Association for the candidates.

The resignation of Dr. Burgess, who had removed from St. Joseph to Louisville, Ky., where he could not attend the meetings of the Association, was tendered and duly accepted.

A paper on the "Injuries of the Flexor Metatarsi" was presented by Dr. R. C. Moore.

Dr. E. J. Netherton: I wish to inquire of the essayist in how long a time he would expect full recovery in these cases, and whether it would be worth while to treat an animal of small value?

Dr. Moore: This would depend upon the extent of the injury. If a simple bruise of the muscular tissue, ten to twelve days would be sufficient; if the tendon be ruptured, or nearly so, it will take much longer. Several cases have been reported where the period of convalescence was ten to twelve months. If the animal be valuable, treatment for a considerable length of time will be justified.

Dr. Heck: This reminds me of a case that Dr. Patterson and I diagnosticated as rupture of the flexor metatarsi. This case recovered in ten days—seemed too rapid to justify the diagnosis. It was a case of a mule used to an express wagon. We got some very fine photographs of the case, but I forgot to bring them with me. It had every symptom of rupture of this tendon or muscle; might possibly have been temporary paralysis of the nerves of this region. The animal could bear the weight of the body on the limb when placed in position, but had no further use of it.

A paper on "Echinococcus Veterinorum" was next presented by Dr. S. Stewart.

Dr. McCurdy: I understood the essayist to say that the adult worm was only found in the intestinal tract of the dog. I wish to inquire if this worm does not sometimes migrate to some other parts of the body?

Dr. Stewart: So far as I am informed no cases have been reported where the adult worm was found in any other part of the body than the intestines. The worm is fixed in its location by its hooks and sucker-disks, and lives by absorption of food intended for its host. The disease is found most frequently in man (in the hydatid form) in Iceland, where the people and dogs closely cohabit.

Dr. McCurdy: Do the records show the disease to be prevalent among Indians and Chinese, where the dog is consumed as food?

Dr. Stewart: The records do not show such prevalency. The parasite is not communicable through eating the flesh of the dog, but is acquired only by the ingestion of the eggs of the adult tapeworm. In countries where the hydatid form of this disease is prevalent among the people it is found that it is also prevalent among the food-producing animals.

Dr. McCurdy: I think of another question. It seems to me that I have seen the cysts very much larger and of a slightly darker color. Do they vary in color?

Dr. Stewart: The cuticular and mother-membranes undergo changes as the hydatids become more and more aged. The cysts become opaque or white, and undergo degeneration, and the original structures become so transformed that it is difficult if not impossible to determine their original character. Often the question is determined by finding the cephalic hooks.

Dr. Cock: Is there any means by which we can diagnosticate this in the hydatid form either in man or animal ante-mortem, and have we any sure means of confirming our diagnosis?

Dr. Stewart: This is certainly a very practical side of the question from the human stand-point. If some of the contents of the cysts be secured by the exploring trocar there is no doubt the proligerous cysts or individual heads would float out with the fluid, and by microscopical examination the character of the cyst would be determined in this way.

Dr. Cock: It seems to me that it is stated that its character may be determined by the absence of albuminous material in the fluid.

Dr. Stewart: I think the absence of albumin is noted in the fluid obtained from a variety of cysts.

Dr. Heck: This discussion concerning the character of the cystic fluid calls to mind our discussion in a former meeting concerning the character of fluid contained in the cystic kidneys, and it might not be out of place for me to revert to the subject here. I have made some analysis of the fluid contained from cystic kidneys, and have found it to be alkaline in reaction; specific gravity 1007; albumin in large quantities. I would be pleased to hear other reports in regard to the cystic kidneys. I find there is a disease or condition in human practice known as hydronephrosis. Whether this is analogous to our cystic kidneys I am not able to say, but the resemblance is very striking. It is supposed that urine has collected through obstruction of the uriniferous tubules or occlusion of the ureters themselves. These cysts do not always contain urine, but sometimes contain fluid of a uriniferous nature.

At the close of the discussion of this paper a recess was taken, that the members might exchange greetings with those who had come in since the opening of the meeting.

Dr. Kelly then presented a paper on "Tuberculosis of Swine."

Dr. S. Stewart: According to the arrangements of the programme it falls to me to open the discussion on the paper just read. I desire to compliment the essayist upon this most excellent presentation of a very interesting subject. He has followed out the disease processes so completely that there is little room for discussion along that line. However, I may be able to offer a few remarks that will stimulate others' thoughts which they will offer in this discussion.

The paper deals very largely with phagocytosis, and, as I understand the theory, it is a correct presentation of the subject. There are some points, however, which I think it would be valuable for us to consider. There is one feature of this theory of phagocytosis which the essayist did not touch upon, and which to me is a most interesting one. I refer to the problem of the manner or process by which the bacillus tuberculosis finds entrance into the solid tissues when introduced into the respiratory or alimentary tracts. It seems that ordinarily the epithelial structures are capable of resisting the invasion unless there be an abrasion of the superficial layers. I have no recollection that bacteriologists claim that the tubercle bacillus has motor power or has the power of migration. There is a theory that the bacillus, probably through a substance which it secretes, is capable of irritating the mucous surfaces and stimulating the phagocyte to reach through the superficial structures and grapple with the bacillus in an endeavor to effect its destruction, and upon withdrawal of the phagocyte the bacillus is carried within the solid tissues. The essayist held that it was only when the lymph-glands had undergone necrotic destruction that the bacilli were carried forward toward the general circulation by enclosing phagocytes. I am inclined to believe phagocytes are capable of and doubtless do escape from tubercular lymph-glands before extensive necrotic changes occur, and that the invasion is usually beyond the point showing gross necrotic lesions. This seems to be true in cases of tubercular invasion of serous membranes. Apparently the tubercular development is in a like stage of progress over large areas of the serous surfaces. If the organism was not carried beyond the point where necrotic changes were plainly visible, then we would find that beginning with the point of invasion the changes would be more complete and more aged than in other parts of the affected membrane. If I remember correctly, it is not often that we can see the various stages of development so plainly marked, showing the gradual advance from one point and spread out over the serous surfaces.

It is probably true that in most cases in swine the infection occurs through the alimentary tract rather than through the respiratory passages, as evidenced by the lesions found in the lungs being apparently of more recent development than those which are found in organs adjacent to the intestinal canal.

I was interested in the statement that swine produce no sputa. Persons who handle swine are well aware that it is difficult to find a bunch of hogs which have been moved to the slaughter-house pens which will not contain a number that do not cough more or less violently when made to move about briskly.

The mucous membrane lining the respiratory tract in swine certainly secretes mucus just the same as like structures in other animals, and I am satisfied that I have observed swine expectorate or throw out mucus when coughing, and I see no reason why the bacilli of tuberculosis might not, by means of sputa, escape from the lungs of swine affected with this disease.

I fully agree with the essayist that if tuberculosis is found in swine the flesh should be condemned and not used for food purposes.

There are a number of gentlemen present who have had large experience in post-mortem examinations of swine who doubtless can contribute materially to this discussion, and I trust they will do so.

Dr. McCurdy: I would like to ask Dr. Kelly if he has noticed what seems to me to be a peculiarity of tuberculosis of the pleura in swine, which is the absence of adhesions between the two pleural surfaces—visceral and parietal—whereas adhesions are quite common in pleural tuberculosis in cattle.

Dr. Kelly: I would say that I have never noticed a case of adhesion of the lung to the chest-wall in cases of tuberculosis in swine. The tubercular nodules seem to be beneath the parietal pleura, causing a bulging at places where the tubercles developed. The tubercles are usually flattened. I have never noticed them to be pedunculated as in the ox.

Dr. McCurdy: I think this point is very important. I have seen a good many cases where the lung is adherent to the thoracic wall and is removed with difficulty; but in tuberculosis the lung is not adherent; it comes out *en masse*.

Dr. Forbes: I think this Association is indebted to Dr. Kelly in bringing this subject to our notice. Tuberculosis in swine is becoming so prevalent that it forces itself upon our attention. We are also indebted to Dr. Kelly for the manner in which he has brought it to our notice, presenting it in such a graphic and interesting way. He has brought the attention of the meeting to the fact that the spleen seems to be a primary seat of the disease. It is easy to account for this, the spleen being an accessory organ of the digestive system, and the abdominal form of tuberculosis being the most frequent in swine. It has been noticed in tuberculosis in cattle that the spleen is seldom if ever affected, the nodules sometimes found on the surface being confined to the capsule, not penetrating the spleen tissue. The difference in the anatomical conformation of the two animals would seem to account for this. In cattle the spleen is attached to the greater curvature of the rumen, and not in close proximity to the true stomach, while in hogs it is almost in direct contact with the true stomach.

In speaking of phagocytosis it was stated by the essayist that the phagocytes seized hold of the bacilli and immediately transported them to the deeper organs to complete their destruction, the spleen being one of the principal organs. During this transportation the bacillus may overcome the phagocyte, the latter becomes sick from its effort to digest the microbe or from the effect of the microbic secretion upon it, and when it reaches the spleen the englobed microbe will be able to liberate itself and proceed to form the nodules characteristic of it.

Another point which interests me was the point brought out by Dr. Stewart regarding the entrance of the bacillus into the tissues. Dr. Kelly, I think, stated in his paper that the phagocytes were of two kinds—microphages and macrophages—the former being white blood-corpuscles and the latter fixed connective-tissue cells. It is possible that the bacilli may be carried by the microphages to the capillaries of the internal organs, where they find conditions favoring their entrance into the tissues. We know that the leucocytes are gifted with movement, evidenced in the process of diapedesis, and one theory of this process is that the leucocytes penetrate between the cells, or that they dissolve the cell-cement, causing the endothelial cells to retract, allowing the corpuscles to pass through readily, and we could suppose that bacilli could be carried through in this way. In the exudate of inflammation we often find large numbers of red globules, which, like the tubercle bacillus, are not gifted with movement, but have escaped through the openings made by the leucocytes. Of course, this is only a theory, and is thrown out in the hope that it will result in stimulating others to an expression of opinion.

Dr. Stewart: The last speaker has shown a way by which the micro-organisms may be transported from the blood-stream into the cellular tissues; he leaves unexplained how the microörganisms get from a mucus surface into the deeper tissues.

The problem of the frequent development of tuberculosis in the structure of the spleen in swine is an interesting one. The essayist has said that the spleen is a source of regeneration of microphages, and it would seem that if a microphage, a sick microphage, succeeded in conveying his burden to the spleen he might get sufficient help to destroy it. I trust that the essayist in his concluding remarks will discuss that point.

Dr. Kelly: While I claim that the spleen has a double function of recuperating disabling microphages, as well as developing new ones, it is probable that sick ones or those which are overcome by the bacilli are disintegrated, liberating the bacilli, and it is in this way they secure a lodgement in the spleen.

Dr. Forbes: Regarding the point of Dr. Stewart, as to how bacilli gains entrance to the tissues from an epithelial surface, I would say that the epithelial cells of a mucus surface, being able to act as phagocytes, could take up the organism, and in the fight which ensues the cell may be overcome and the microbe get deeper into the tissues.

Dr. Cock: There might be one question arise as to how the bacillus enters the mucous membrane. Under certain conditions we may find mucus from the lungs of the human which contains bacilli, while they are not affected with the disease at all. In order for the germs to enter there must be a lowering of the vitality of the mucous membrane. I believe that the way the bacillus enters the mucous membrane it throws out a substance or ptomaïn which destroys the connecting substance between the cells, and in that way finds passage through the mucous membrane.

Dr. Kelly: I had always thought that the germs or spores of tuberculosis were passed through the intestinal mucous membrane by osmosis during the process of absorption, in this way gaining entrance to the lacteals, and are carried to the lymph-channels. It is generally understood that the disease spreads in one way by contiguity of tissues; after gaining entrance to a tissue or organism it may spread from the centre by its own growth or prolongation.

Dr. Stewart: Before closing this subject it might not be amiss to suggest that the organism being a vegetable may vegetate and extend prolongation through the superficial structures of the mucous membrane, and in this way may find entrance into the deeper tissues. Some writers have held that the spores were small enough to be carried in during the process of osmosis.

Dr. B. F. Kaupp presented a paper on "Leukæmia."

Dr. Sihler: I have seen quite a number of cases of leukæmia, and was fortunate enough to treat a case in a dog. I want to state that I was not certain what the malady was when I was treating the case. The symptoms manifested were languor, loss of appetite, constipation, and an irregular pulse. On examination I found what I thought was a tumor in the abdominal cavity. I gave a laxative, then a second dose. The next day the dog died. Upon post-mortem I found the spleen was two feet long, six inches wide, and two inches thick, and then I did not know exactly what I had until I began to look the matter up. It was the first case of leukæmia I had ever seen. I have seen two or three cases in calves. In several cases that I have had the opportunity of seeing the lymphatic glands were of a dark color and varied in size from a hen-egg to a goose-egg. I have seen several cases in hogs, and one or two cases where the lymphatics were dark in color and the spleen very much enlarged.

Dr. Moore: Did you find any symptoms of anæmia in the dog?

Dr. Sihler: The attack seemed to come on very suddenly. Was sick only a day or two before I was called. The dog was in good flesh.

Dr. McCurdy: I saw a case in a steer that was in very good condition where this disease was diagnosed. All the lymphatics were very much enlarged, very nearly all the same size, about as large as a man's fist, and the lesions were diagnosticated as those of leukæmia. This case was diagnosticated as such by Dr. Adair, who has had a great deal of experience among cattle, and was confirmed by Dr. Bennett. The spleen was enlarged to about three times its normal size.

Dr. Sihler: I remember a case in a cow diagnosticated ante-mortem as actinomycosis, but post-mortem proved it to be leukæmia.

Dr. Heck: I have been very much interested in this paper. Before coming to the meeting I made it a point to look up the literature on this subject, but found but little in our veterinary works; more, perhaps, in Zuill's translation of Friedberger and Fröhner's *Pathology and Therapeutics of Domestic Animals* than in any other book. I had recourse to some works on human medicine, and I was particularly struck with the classification of blood diseases in one particular work,[1] and thought it was worth presenting to the Association. The essayist has laid no particnlar stress on the fact of leukæmia being a blood disease, nor has he made any classification of varieties of this disease. The human practitioner makes a distinction of several conditions, which we group together with leukæmia; therefore I note the following classification, which I think is a most excellent one:

1. Anæmia.
   *a*. Primary anæmia.
      1. Chlorosis.
      2. Pernicious anæmia.
   *b*. Secondary anæmia.
2. Leukæmia. Splenic myelogenous; lymphatic.
3. Leucocytosis.
4. Hodgkin's disease.

This classification is based purely on the condition of the blood, and is made possible only by a system of staining (Ehrlich's triple stain) when an

[1] American System of Practical Medicine, pp. 633–702.

altered or diseased condition of the white and red blood-cells, particularly the former, is observed. We find a peculiar blood phenomena existing in each of the outlined conditions; for instance, in pernicious anæmia we find a certain overgrown or enlarged red, nucleated cell (megaloblast), which is characteristic of the trouble, while all the red cells are often enlarged. In normal blood we find several varieties of white cells, the principal ones called polymorphonuclear neutrophiles, 60 to 70 per cent., and lymphocytes, 20 to 30 per cent. In leukæmia we have two varieties of the disease —the splenic and the lymphatic. The splenic is called splenic-myeologenous, owing to a very much enlarged white cell, which Prof. Ehrlich calls a myelocyte, and it seems to be the predominating cell. In the lymphatic form the blood is filled with small lymphocytes about the size of red cells. Leucocytosis is simply an increase in the normal white cells, and may be either physiological or pathological. Hodgkin's disease is known as pseudo-leukæmia, splenic anæmia, lymphoma, etc. We know it by the term lymphoma, and it is characterized by a general enlargement of all the lymphatic glands and sometimes the spleen. There is no perceptible increase in the white cells, and it is only distinguished from leukæmia by a blood examination. Of course, the etiology of these diseases is unknown, and with your permission I will read a short extract from the author commenting on this classification: "All our knowledge of the so-called blood diseases is of the most fragmentary and unsatisfactory nature, and all our statements concerning them must therefore be considered as in the highest degree provisional and open to revision as soon as new light appears. The origin of the blood and the method of its reproduction and renewal are matters worthy of speculation. We are ignorant of whether there are any diseases of the blood itself, except the parasitic diseases, like malaria, and whether morbid blood changes are causes or results of other organic lesions. We may doubt whether the changes both in the blood and in the (supposed) blood-making organs are not both of them due to some third factor, itself unknown. Finally, the identity and individuality of the several blood diseases are open to considerable doubt. In a few years we may have changed all that. Pernicious anæmia and leukæmia may be found to be only different types of a single disease. Chlorosis may be classed with the other secondary anæmias, and Hodgkin's disease may be grouped with the malignant tumors or infectious diseases. Still, we must keep to some nomenclature while a better is being evolved, and the following division of the morbid phenomena to be considered must serve us for the present."

Dr. Stewart: This classification is quite an interesting addition to the discussion. I noticed the essayist did not enter into the blood phase of the disease or into its etiology, but only took up the gross pathological lesions as he found them. The several diseases mentioned in this classification just quoted are likely to come to the attention of the examiner of animals. Very few veterinarians stop to make any distinction as to the form of anæmia. I could readily see how one who did not make any study of the blood phase could mistake Hodgkin's disease for leukæmia. The last speaker raised the question of the essayist not making any examination of the blood to make sure the cases were leukæmia. I wish to say that in the case the essayist cited as reported by myself an examination of the blood was made and it was found that there was one white blood-cell to twenty red ones.

Dr. Heck: I wish to beg the gentleman's pardon if I misunderstood him, and I assure him nothing has been said to cast any reflection on his paper. He has dealt with the clinical aspect of the disease almost entirely, and I congratulate him on having seen so many cases. I simply mentioned this classification to show how much more thoroughly the subject has been gone into by human practitioners and how far behind we seem to be. I am not sure that we find analogous conditions in our practice, but I should be very glad if some of our members would make some blood-counts of properly stained specimens. No one has mentioned the length of time these patients live. I will say in human subjects they seldom live longer than three or four years, the splenic-myelogenous form being much more chronic than the lymphatic variety. Persons suffering from Hodgkin's disease may live an indefinite period.

A clinic being a part of the programme attracted considerable interest, which it is hoped will grow in favor with future meetings and prove to be an attraction to practitioners; that it will increase our attendance and promote the interest in our Association work.

The operations performed were as follows: Neurectomy (lower operation), Dr. Moore. Operation for the cure of the cribbing habit, excision of a portion of the sternothyrohyoideus muscle, and resection of the motor branch of the spinal accessory nerve supplying the sternomaxillaris muscle, anæsthesia being produced by intravenous injection of chloroform, Dr. Moore. Peroneal tenotomy for stringhalt, Dr. Simpson. Cunean tenotomy for spavin-lameness, Dr. Patterson. Peroneal tenotomy, Dr. Steel. Ovariotomy (bitch), Dr. Moore. Ovariotomy (bitch), Dr. Black.

Dr. Stewart: By provision of the By-Laws the next meeting should be held in this city; but in compliment to our friends from St. Joseph, who have so generously turned out to attend this meeting, I move that when we adjourn that the By-Laws be suspended and we adjourn to meet in St. Joseph. The motion was duly seconded and carried. Dr. Heck spoke in regard to subscriptions to the various veterinary journals. He states that all the veterinarians in St. Joseph except two are subscribers to these journals, a claim which cannot be made by any other city.

On motion of Dr. Forbes, a vote of thanks was extended to the essayists of the evening.

It was moved and carried that a vote of thanks be extended to the Kansas City Veterinary College for the use of its rooms for this meeting.

On motion, the Association adjourned.

W. A. Heck,
Secretary.

